# Intranasal soluble ACE2 improves survival and prevents brain SARS-CoV-2 infection

**DOI:** 10.26508/lsa.202301969

**Published:** 2023-04-11

**Authors:** Luise Hassler, Jan Wysocki, Jared T Ahrendsen, Minghao Ye, Ian Gelarden, Vlad Nicolaescu, Anastasia Tomatsidou, Haley Gula, Cosimo Cianfarini, Peter Forster, Nigar Khurram, Benjamin D Singer, Glenn Randall, Dominique Missiakas, Jack Henkin, Daniel Batlle

**Affiliations:** 1 Division of Nephrology/Hypertension, Department of Medicine, Northwestern University , Feinberg School of Medicine, Chicago, IL, USA; 2 Charité Universitätsmedizin Berlin, Berlin, Germany; 3 Department of Pathology, Northwestern University , Feinberg School of Medicine, Chicago, IL, USA; 4 Department of Microbiology, University of Chicago, Chicago, IL, USA; 5 Ricketts Regional Biocontainment Laboratory, University of Chicago, Lemont, IL, USA; 6 Division of Pulmonary and Critical Care Medicine, Northwestern University , Feinberg School of Medicine, Chicago, IL, USA; 7 Center for Developmental Therapeutics, Northwestern University , Evanston, IL, USA

## Abstract

A soluble ACE2 protein bioengineered for long duration of action and high affinity to SARS-CoV-2 was administered either intranasally (IN) or intraperitoneally (IP) to SARS-CoV-2–inoculated k18hACE2 mice. This decoy protein (ACE2 618-DDC-ABD) was given either IN or IP, pre- and post-inoculation, or IN, IP, or IN + IP but only post-inoculation. Survival by day 5 was 0% in untreated mice, 40% in the IP-pre, and 90% in the IN-pre group. In the IN-pre group, brain histopathology was essentially normal and lung histopathology significantly improved. Consistent with this, brain SARS-CoV-2 titers were undetectable and lung titers reduced in the IN-pre group. When ACE2 618-DDC-ABD was administered only post-inoculation, survival was 30% in the IN + IP, 20% in the IN, and 20% in the IP group. We conclude that ACE2 618-DDC-ABD results in markedly improved survival and provides organ protection when given intranasally as compared with when given either systemically or after viral inoculation, and that lowering brain titers is a critical determinant of survival and organ protection.

## Abstract (precis)

A soluble ACE2 protein bioengineered to have extended duration of action and enhanced affinity for SARS-CoV-2 provides markedly improved survival and organ protection when administered intranasally. Reducing brain SARS-CoV-2 titers is an important determinant of therapeutic efficacy.

## Introduction

Early in 2020, shortly after ACE2 was reported to be the main cell entry receptor for SARS-CoV-2 (1, 2), our laboratory proposed the use of soluble ACE2 proteins to neutralize SARS-CoV-2 via a decoy effect (3). The potential of soluble ACE2 proteins to neutralize SARS-CoV-2 was soon after shown using human organoids (4). This cellular model expresses human ACE2, the essential cell entry receptor for SARS-CoV-2 and TMPRSS2, a protease critical for internalization of the ACE2–SARS-CoV-2 complex (2, 4, 5, 6). Because mice and rats are resistant to SARS-CoV-2, the human transgenic k18hACE2 mouse has been used widely to test the efficacy of new interventions geared to prevent and treat SARS-CoV-2 infection (7, 8, 9, 10, 11, 12, 13, 14, 15). The k18hACE2 model is lethal when infected with a high dose of WT SARS-CoV-2 and replicates severe lung disease in humans (10, 11, 14, 16, 17). There is also some evidence of brain injury (9, 10, 14, 18, 19, 20), but the precise cause of the universal lethality is not known. Expression of ACE2 in brain neurons has been demonstrated by immunocytochemistry and enzymatic assays suggesting that neuroinvasion of SARS-CoV-2 may occur (21, 22).

Soluble ACE2 proteins for SARS-CoV-2 offer theoretical advantages over antibody-based approaches which are increasingly resistant to emerging SARS-CoV-2 variants (23, 24, 25, 26, 27, 28, 29, 30, 31, 32, 33). For instance, multiple passaging in the presence of soluble ACE2 proteins does not lead to mutational escape of SARS-CoV-2, whereas mutational escape of the virus is seen rapidly after passaging in the presence of monoclonal antibodies (34). ACE2 decoys have a unique advantage over monoclonal antibodies because viral mutants are unlikely to decrease decoy affinity without simultaneous loss of ACE2 affinity, making decoys less susceptible to resistance by viral mutation (35, 36, 37).

We bioengineered a soluble ACE2 protein, based on a truncate of human ACE2 with 618 amino acids that was fused with an albumin-binding domain (ABD) to confer prolonged in vivo duration of action via albumin binding (5). Later, we used a dodecapeptide (DDC) motif (38) to form a dimer and were able to enhance the binding affinity for SARS-CoV-2 markedly (8). In the k18hACE2 model infected with SARS-CoV-2, administration of this protein (termed ACE2 618-DDC-ABD) resulted in markedly improved survival and greatly reduced lung injury (8). ACE2 618-DDC-ABD in this previous study was administered combined intranasally (IN) and intraperitoneally (IP) to ensure proof-of-concept efficacy but the brain histopathology was not studied (8). Here, we investigated the intranasal as compared with the intraperitoneal administration of ACE2 618-DDC-ABD and in addition examined the impact of treatment when initiated before or only after viral inoculation on survival, organ protection, and viral titers.

## Results

### Survival, clinical score, and weight loss in SARS-CoV-2–infected k18hACE2 mice

**Figure 1.**
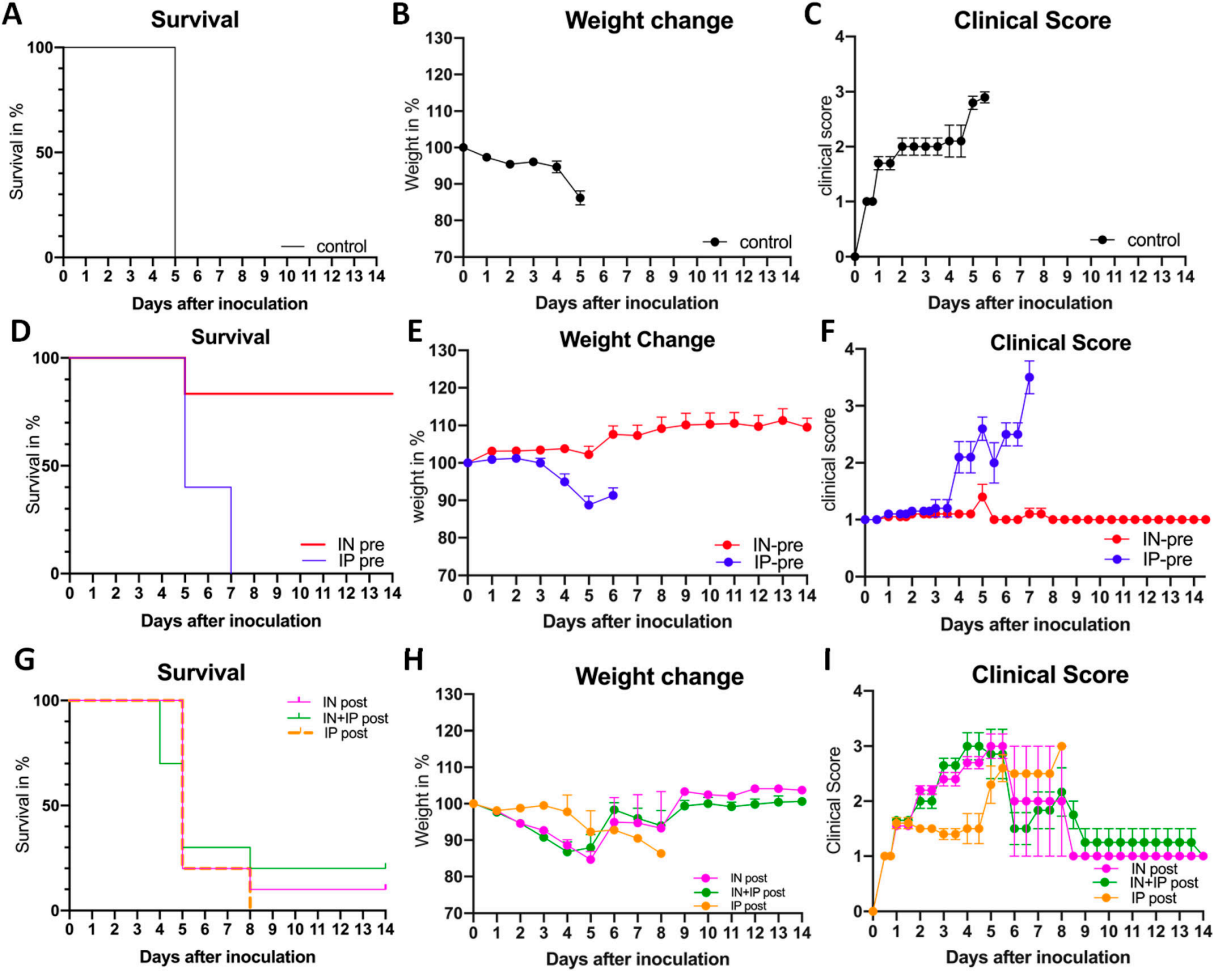
Survival, body weight, and clinical score after viral inoculation with 2 × 10^4^ PFU SARS-CoV-2 comparing intranasal (IN) versus intraperitoneal (IP) versus IN + IP administration of ACE2 618-DDC-ABD to k18hACE2 mice 1 h before and 24 and 48 h post (pre group, D, E, F) or only 24, 48, and 72 h post (post group, G, H, I). **(A, B, C)** Vehicle-treated group. Infected mice that received vehicle (BSA in PBS, black) had 0% survival on day 5 (A), lost up to 20% of their body weight (B), and developed high clinical scores (C). **(D, E, F)** Administration before and post-viral inoculation. In the IN-pre group (red), nine out of 10 mice survived until day 5 (90%), whereas in the IP-pre group (blue) only four out of 10 mice survived until day 5 (40%). Four of the nine surviving mice from the IN-pre group that were healthy by clinical score were then euthanized to obtain organs for comparison, and the remaining five mice all survived until day 14. By contrast, none of the four remaining mice in the IP-pre group survived until day 14 (D). The IN-pre group (red) had no body weight loss (E), and clinical score was normal (F), whereas the IP-pre group (blue) experienced body weight loss and worsening clinical score. **(G, H, I)** Administration post-viral inoculation. In the IN + IP-post group (green), 3 out of 10 mice survived until 5 (30%) and 2 out of 10 (20%) until day 14. In the IN-post group (pink), survival was 2 out of 10 (20%) on day 5 and 1 out of 10 (10%) on day 14 (G). In the IP-post group (orange), survival was 1 out of 5 (20%) on day 5 and 0 out of 5 (0%) on day 14 (G). In most mice that received ACE2 618-DDC-ABD post-viral inoculation weight loss was severe, and clinical scores were high although a few mice had stable weight and normal clinical score (H, I).

The effects of intranasal (IN) versus intraperitoneal (IP) administration of ACE2 618-DDC-ABD were examined in the k18hACE2 mouse, a lethal model of SARS-CoV-2 infection. According to study protocol, animals that lost more than 20% of their body weight or had a clinical score of three or higher were humanely euthanized, and this was considered a mortality event (7, 8). Survival was 0% in the infected untreated control mice, all of which had to be humanely euthanized on day 5 (Fig 1A). They all had severe body weight loss (Fig 1B) and/or a high clinical score (Fig 1C).

### Administration of ACE2 618-DDC-ABD pre- and post-viral inoculation

In mice that received ACE2 618-DDC-ABD combined pre- and post-viral inoculation, survival on day 5 was 90% in the IN-pre group (9 out of 10), and only 40% in the IP-pre group (4 out of 10) (*P* = 0.0024) (Fig 1D). As compared with the infected untreated group with 0% survival, the IN-pre group survival was also highly significant (*P* = 0.0084). In the IP-pre group, survival was improved but it did not reach statistical significance as compared with the infected untreated group (*P* = 0.1106, all by log-rank [Mantel–Cox] test).

To obtain organs for comparison, four of the nine mice from the IN-pre group that were not affected by SARS-CoV-2 inoculation (by body weight and clinical score) were euthanized on day 5; the remaining five all survived until the end of the study (day 14) with near normal clinical scores and no weight loss (Fig 1E and F). The mice in the IP-pre group, by contrast, all had to be euthanized by day 7 because of worsening clinical scores and weight loss, according to the study protocol approved by the Institutional Animal Care and Use Committees (IACUC) (see the Materials and Methods Section) (Fig 1D–F).

### Administration of ACE2 618-DDC-ABD only post-viral inoculation

In mice that received ACE2 618-DDC-ABD only post-viral inoculation, survival in the IN + IP-post group was 30% on day 5 (3 out of 10 mice) and 20% on day 14 (2 out of 10 mice) (Fig 1G). In the IN-post group, survival was 20% on day 5 (2 out of 10) and 10% on day 14 (1 out of 10). In the IP-post group, survival was 20% on day 5 (1 out of 5 mice) but 0% on day 14 (0 out of 5 mice) (Fig 1G). For comparison, infected untreated mice had 0% survival on day 5 (Fig 1A). Most of the animals that received ACE2 618-DDC-ABD post-viral inoculation had rapid body weight loss and a worsening clinical score, but some (n = 2 IN + IP-post, n = 1 IN-post) recovered over the course of the study and survived until day 14 with stable body weight and relatively good clinical scores (Fig 1H and I).

As compared with untreated infected controls, in the post-inoculation groups (IN + IP-post, IN-post, and IP-post), survival was improved, but the differences did not reach statistical significance (*P* = 0.8171, *P* = 0.2994, and *P* = 0.3173, respectively). These three posttreatment groups (IN + IP-post, IN-post, and IP-post) had significantly worse survival than the IN-pretreatment group (*P* = 0.0181, *P* = 0.0046, and *P* = 0.0064, respectively). When compared with the IP-pretreatment group, there were no statistically significant differences in survival for any of these three posttreatment groups (*P* = 0.8237, *P* = 0.7827, and *P* = 0.8914, respectively).

### SARS-CoV-2 brain and lung titers

**Figure 2.**
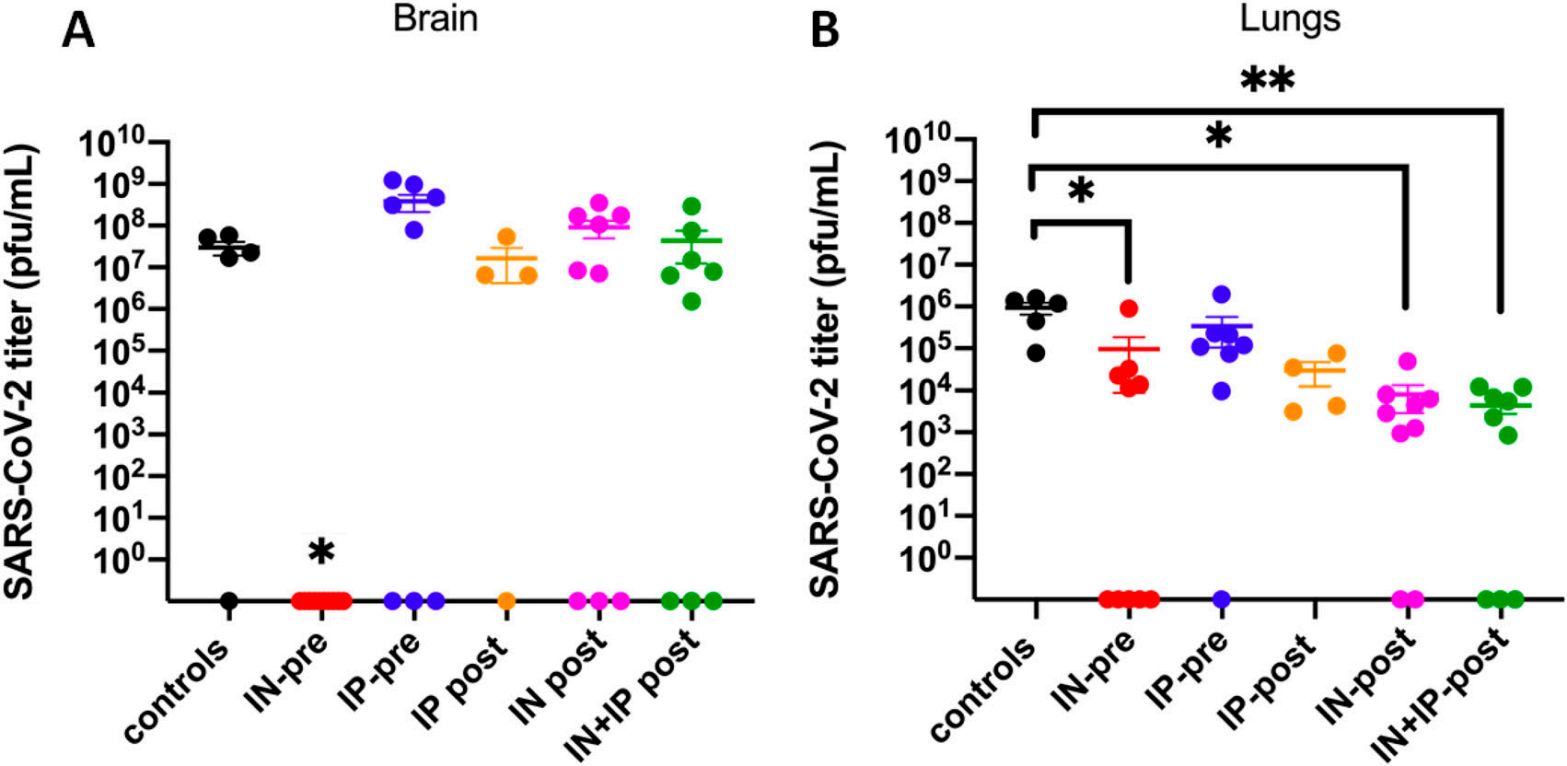
Brain (A) and lung (B) viral titers in k18hACE2 mice inoculated with 2 × 10^4^ PFU SARS-CoV-2 that received ACE2 618-DDC-ABD 1 h pre- and 24 and 48 h post-viral inoculation. **(A)** Brain titers were high in all but one infected untreated control mice (black), most mice from the IP-pre group (blue) and the post-treated groups (orange, pink, green). In the IN-pre group (red), by contrast, titers were undetectable in all mice, and significantly lower than that in the IP-pre group (*P* = 0.0167). **(B)** Lung titers were highest in the untreated infected control mice (black). In the other groups, lung titers were lower and non-detectable in some mice. As indicated by the asterisks, significant differences were found between controls (black) and the IN-pre group (red, *P* = 0.0244), the IN-post group (pink, *P* = 0.0136), and the IN + IP-post group (green, *P* = 0.0078). Note that in two mice from the IP-pre group and one mouse per post-treated group organs could not be obtained. Significance was calculated using ANOVA followed by Dunn’s multiple comparisons test. If not indicated by the asterisk, the differences did not reach statistical significance. Viral titers were normalized by organ weight. Mean ± SEM are shown.

Brain (Fig 2A) and lung viral titers (Fig 2B) were very high in infected untreated mice (3.0 × 10^7^ ± 1.1 × 10^7^ PFU/ml and 9.33 × 10^5^ ± 2.87 × 10^5^ PFU/ml, respectively). When comparing the two organs, the titers were significantly higher in the brain than lung tissue (*P* = 0.0295). Brain titers in the IN-pre–treated group were undetectable (0 ± 0 PFU/ml), whereas titers in the IP group were very high (3.82 × 10^8^ ± 1.69 × 10^8^ PFU/ml, *P* = 0.0167) and similar to the infected untreated group (Fig 2A).

In all post-treated groups, brain viral titers were high or decreased only marginally as compared with the untreated infected mice (IP-post: 1.66 × 10^7^ ± 1.25 × 10^7^; IN-post: 9.06 E × 10^7^ ± 4.08 × 10^7^; and IN + IP-post: 4.4 × 10^7^ ± 3.17 × 10^7^) (Fig 2A). In the few survivors from the IN + IP-post and IN-post groups (n = 2 and n = 1, respectively), however, brain titers were undetectable on day 14 (Fig 2A).

Lung titers were lower in all pre- and post-treated groups as compared with the infected untreated mice and reached statistical significance for the IN-pre, IN-post, and IN + IP-post groups (Fig 2B). The IN-pre group had lower titers than the IP-pre group, but the difference did not reach statistical significance (9.67 × 10^4^ ± 8.79 × 10^4^ and 3.37 × 10^5^ ± 2.32 × 10^5^ PFU/ml, respectively, *P* = 0.3615) (Fig 2B).

In the post-treated groups, the lung titers were as follows: IP-post: 2.94 × 10^4^ ± 1.71 × 10^4^ PFU/ml; IN-post: 8.06 × 10^3^ ± 5.17 × 10^3^; and IN + IP-post: 4.4 × 10^3^ ± 1.66 × 10^3^(Fig 2B). The IN-pre, IN-post, and IN + IP-post groups had significantly lower viral titers than the infected untreated group (*P* = 0.0244, *P* = 0.0136, and *P* = 0.0078, respectively), whereas the differences between each other did not reach statistical significance. When ACE2 618-DDC-ABD was given only intraperitoneally (either pre- or post-viral inoculation), lung titers were not significantly reduced.

### Brain histopathology

**Figure 3.**
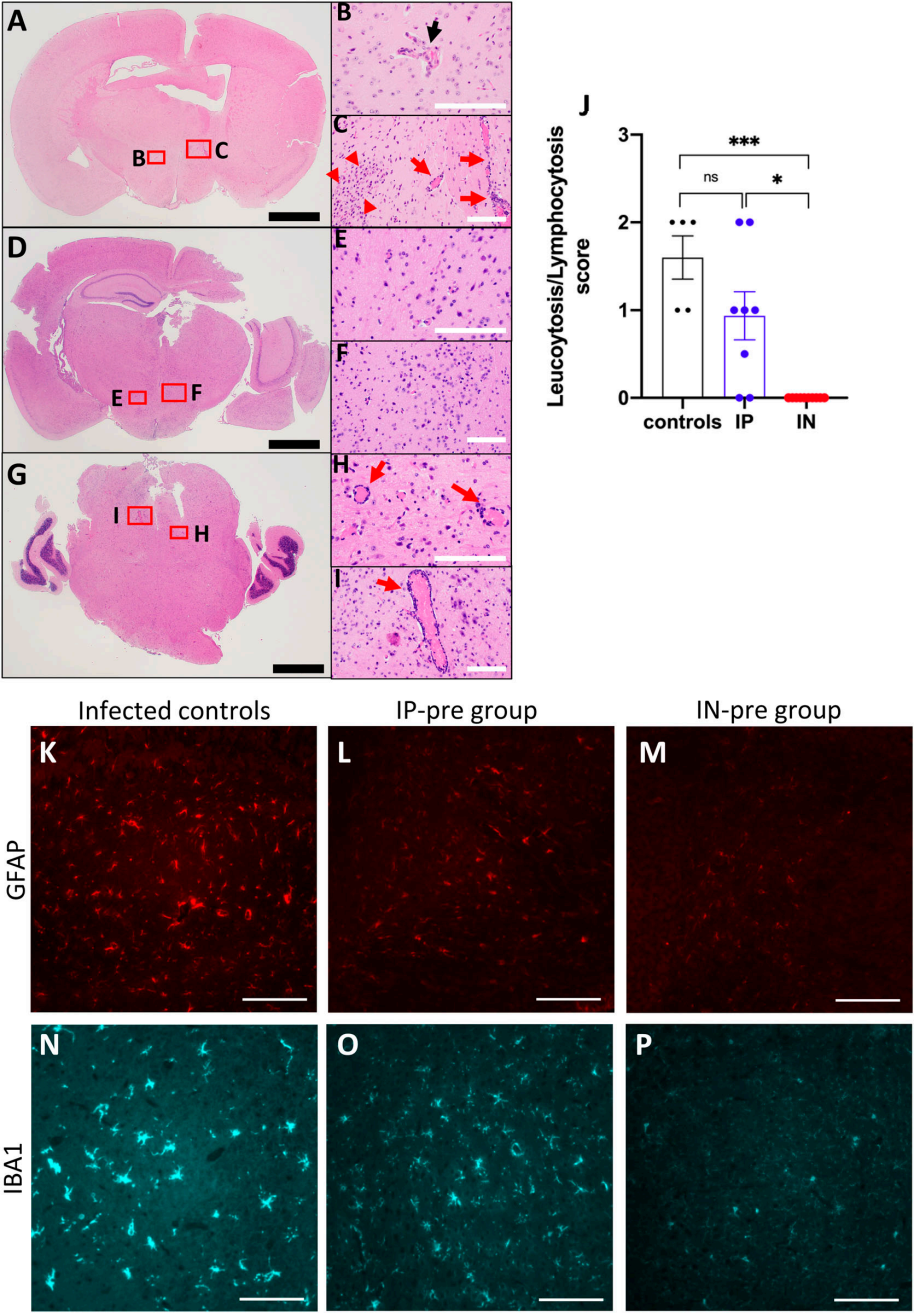
Representative neuropathology of k18hACE2 mice inoculated with 2 × 10^4^ PFU SARS-CoV-2. **(A, B, C)** Example of an infected untreated mouse with endothelial hypertrophy (black arrow, (B)), perivascular (red arrows, (C)), and parenchymal lymphocytosis (red arrowheads, (C)) in the hypothalamus. **(D, E, F)** Example of a mouse from the IN-pre group that received ACE2 618-DDC-ABD showing normal appearing hypothalamus, including vasculature. Black scale bars = 1 mm, white scale bars = 100 μm. **(G, H, I)** Example of a mouse from the IP-pre group that received ACE2 618-DDC-ABD also showing perivascular lymphocytosis (red arrows, (H, I)) in areas of the brainstem. **(J)** When data from all animals (n = 5 controls, n = 8 IP-pre, n = 10 IN-pre) were scored for leukocytosis/lymphocytosis, there were significant differences between the groups (*P* = 0.0007 and *P* = 0.01, respectively). Data shown as mean ± SEM. Significance was calculated by one-way ANOVA followed by Dunn’s multiple comparisons test. **(K, L, M)** Examples of GFAP staining (red) which is strongest in an untreated infected brain (K), reduced partially in a brain from the IP-pre group (L), and very weak in a brain from the IN-pre group (M). **(N, O, P)** Examples of IBA1 staining (blue) which is strongest and showed the most pronounced ramifications in an untreated infected brain (N), reduced partially in a brain from the IP-pre group (O), and almost completely absent in a brain from the IN-pre group (P). All IF-photomicrographs were taken at 40x magnification, scale bar = 100 μm.

In brains of the infected untreated mice, leukocytosis and/or endothelial hypertrophy were features consistently seen, although of variable degrees. These findings were mainly observed in the striatum, cerebral cortex, and hypothalamus (Fig 3A–C). In addition, both perivascular and parenchymal inflammation were occasionally seen in the hypothalamus and basal ganglia. In the IN-pre group, by contrast, these histopathologic features in hypothalamus and cortex were absent in all mice (Fig 3D–F). In the IP-pre group, there was also perivascular leukocytosis in areas of the brainstem in some (Fig 3G–I) but not all mice. The leukocytosis score was improved significantly for the IN-pre group compared with both infected untreated mice and the IP-pre group (*P* = 0.0007 and *P* = 0.0199, respectively) (Fig 3J).

Another histopathologic abnormality found in untreated infected brains was neuronal pyknosis. This was scored on a scale of 0–3 and was significantly reduced in both the IP-pre (0.875 ± 0.125) and IN-pre group (1 ± 0) as compared with untreated infected mice (2 ± 0, *P* = 0.0001 and *P* = 0.0003, respectively).

**Figure S1.**
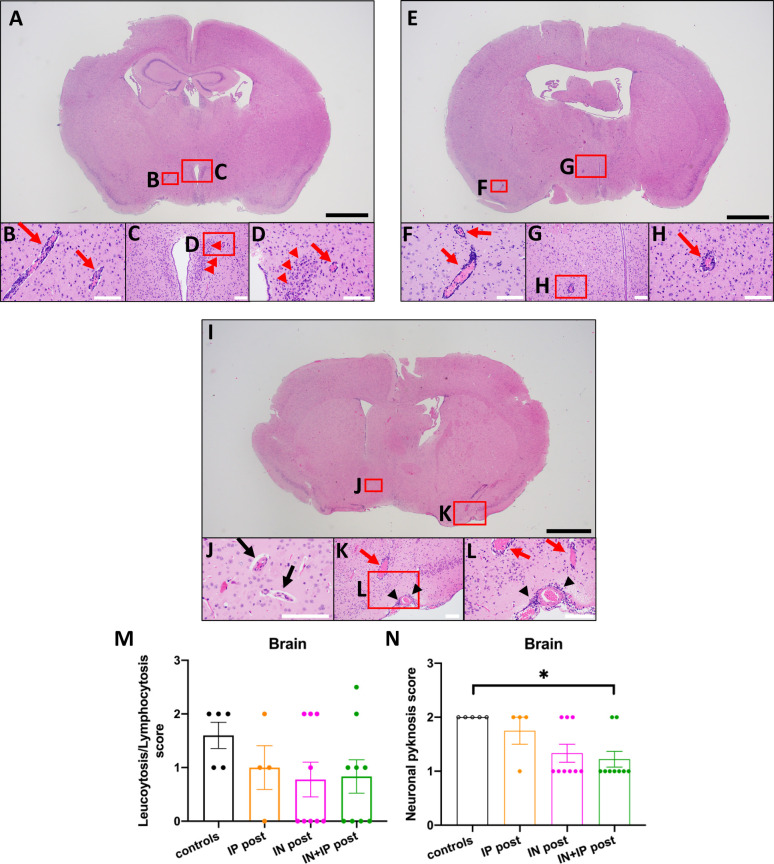
Representative neuropathology of k18hACE2 mice inoculated with 2 × 10^4^ PFU SARS-CoV-2 that received ACE2 618-DDC-ABD post viral inoculation. **(A, B, C, D)** In a mouse from the IP-post group that received ACE2 618-DDC-ABD, perivascular lymphocytosis (red arrows) and parenchymal inflammation (red arrowheads) were seen in the hypothalamus (B, C, D). **(E, F, G, H)** In a mouse from the IN-post group, perivascular lymphocytosis (red arrows) was seen in the lateral cortex (F) and hypothalamus (G, H). **(I, J, K, L)** In a mouse from the IN + IP-post group, endothelial hypertrophy was seen in the hypothalamus ((J), black arrows). The lateral cortex showed perivascular lymphocytosis ((K), red arrows) and leptomeningeal inflammation ((K, L), black arrowheads). Black scale bars: 1 mm; white scar bars: 100 μm. **(M)** The histopathological score for leukocytosis/lymphocytosis was the highest in infected untreated controls (black). It was decreased in all treated groups (color), but these differences were not statistically significant. **(N)** The histopathological score for neuronal pyknosis was highest in infected untreated controls (black). It was lower in the IP-post group (orange), the IN-post group (pink), and IN + IP-post group (green). The differences between the groups were statistically significant for the IN + IP-post group (green) as compared with controls (*P* = 0.0370). Mean ± SEM are shown. Significance was calculated by one-way ANOVA followed by Dunn’s multiple comparisons test.

In the IP-post group, perivascular/parenchymal inflammation and endothelial hypertrophy in the hypothalamus was also seen (Fig S1A–D). Likewise, in the IN-post and IN + IP-post groups, leptomeningeal and perivascular lymphocytosis and endothelial hypertrophy in the hypothalamus and lateral cortex were seen in some animals (Fig S1E–L) similar to the infected untreated mice (Fig 3A–C). The score for perivascular leukocytosis was lower in the IP-post, IN-post, and IN + IP-post group than in the infected untreated mice but did not reach statistical significance (Fig S1M). The score for neuronal pyknosis was decreased in all post-treated groups, but the difference was significant only for the IN + IP-post group as compared with infected untreated mice (*P* = 0.037) (Fig S1N).

### Immunofluorescence for markers of astrocytes and microglia

**Figure S2.**
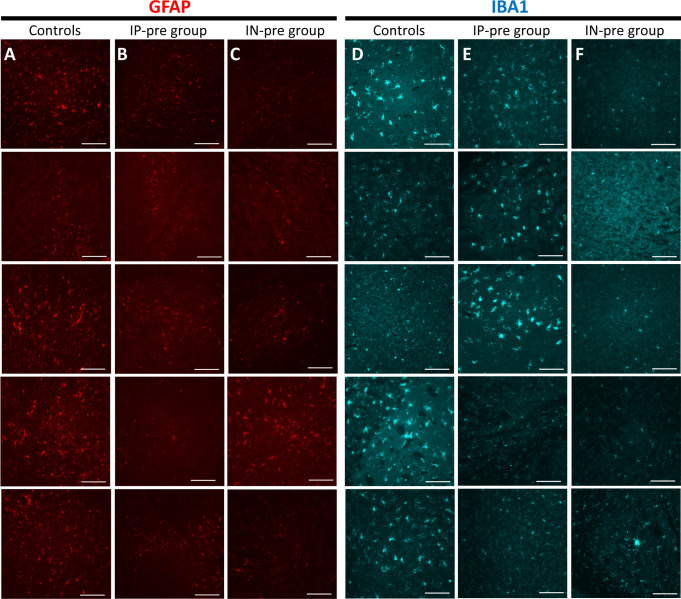
Representative examples of GFAP and IBA1 immunofluorescence staining in the brains of k18hACE2 mice inoculated with 2 × 10^4^ PFU SARS-CoV-2. ** (A, B, C)** GFAP staining (red) is strong in the brain of five infected untreated controls (A), whereas it is partially reduced in the brain of five mice from the IP-pre group that received ACE2 618-DDC-ABD (B). **(C)** In 5 mice from the IN-pre group, GFAP staining is further reduced in some mice (C). **(D, E, F)** IBA1 staining (blue) is strong in untreated infected controls (D) and mice from the IP-pre group that received ACE2 618-DDC-ABD (E). **(F)** In mice from the IN-pre group, by contrast, IBA1 staining is reduced (F). All photomicrographs were taken at 40x magnification, scale bar = 100 μm. Please note that the images of the upper six panels of Fig S2 are the same as the examples shown in Fig 3K–P, but in Fig S2 we show four more examples per group (five animals per group total).

Immunofluorescence for the astrocyte marker GFAP and the microglial marker IBA1 revealed high expression of both markers in infected untreated mice in patterns consistent with reactive astrocytosis and microgliosis, respectively (Fig 3K and N). GFAP staining was partially decreased in the IP-pre group (Fig 3L) and markedly reduced in the IN-pre group (Fig 3M). IBA1 staining showed partially reduced microglia cells with ramifications in mice in the IP-pre group (Fig 3O), whereas the ramifications were markedly reduced in the IN-pre group (Fig 3P). Several other examples of these differences are shown in the supplement (Fig S2).

### Lung histopathology

**Figure 4.**
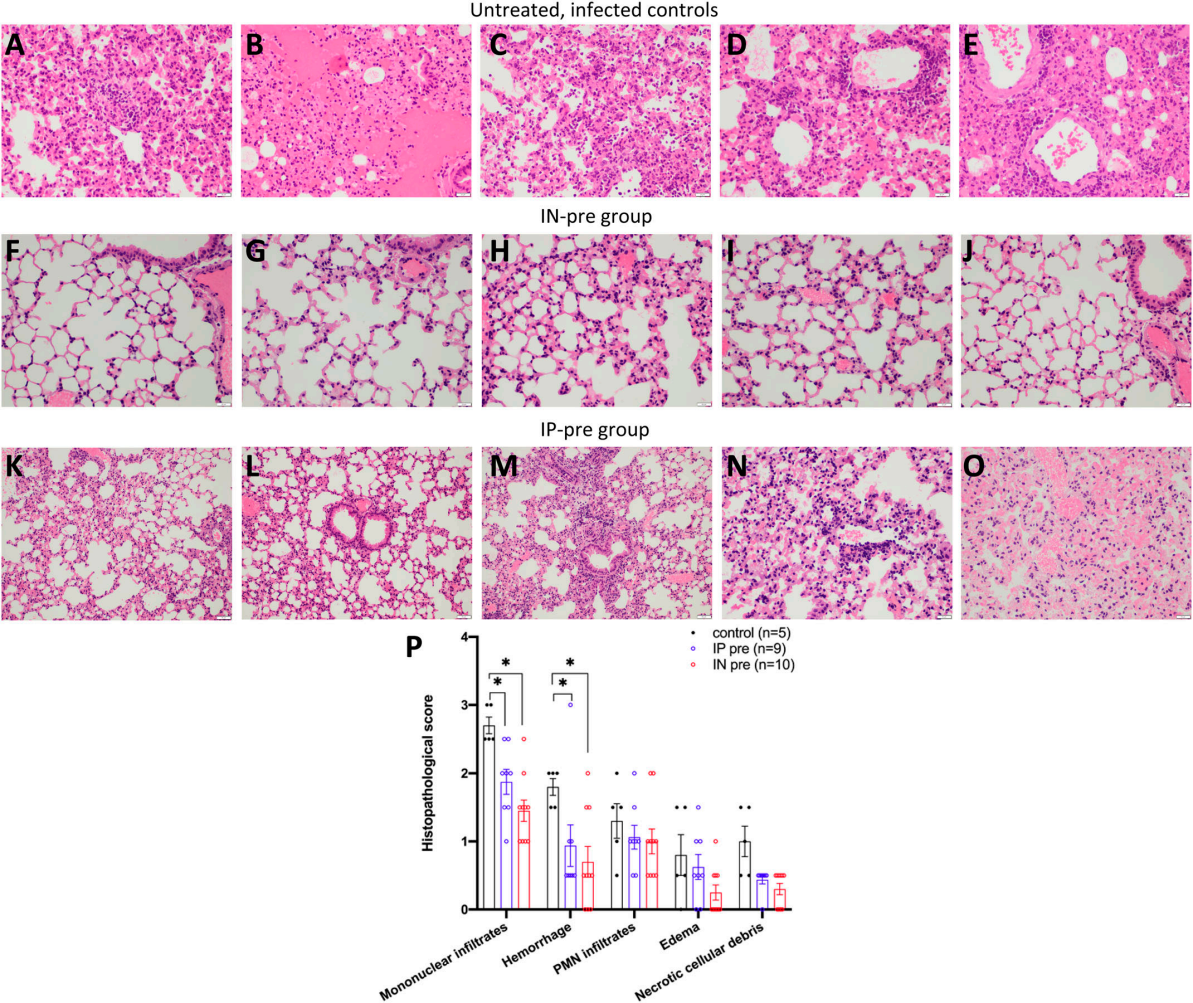
Representative examples of lung histopathology in H&E-stained lung slides of SARS-CoV-2–infected k18hACE2 mice. **(A, B, C, D, E)** Five different mice from the vehicle control group (n = 5) that were infected with SARS-CoV-2 show dense perivascular mononuclear infiltrates (D, E), collections of intra-alveolar neutrophils (C), rare foci of necrotic debris (A), and alveolar hemorrhage (B). **(F, G, H, I, J)** Five mice from the IN-pre group that received ACE2 618-DDC-ABD (n = 10) show near normal lung histopathology with minimal perivascular mononuclear infiltrates and hemorrhage in some cases. **(K, L, M, N, O)** Five mice from the IP-pre group that received ACE2 618-DDC-ABD (n = 8) show milder perivascular mononuclear infiltrates (M, N) with other areas of near normal lung histopathology (K, L), focal minimal alveolar hemorrhage (O), and occasional intra-alveolar neutrophils. **(P)** The lung histopathology scores for mononuclear infiltrates, hemorrhage, PMN infiltrates, edema, and necrotic cellular debris are high in the controls (black) and lower in both the IP-pre (blue) and IN-pre (red) groups. Data shown as mean ± SEM are shown. Significance is indicated in the figure with * = *P* < 0.05, calculated by mixed-effects analysis followed by Tukey’s multiple comparisons test. All photomicrographs were taken from H&E-stained sections at 40x magnification, scale bar = 500 μm.

Lungs from untreated infected mice showed dense perivascular mononuclear infiltrates and collections of intra-alveolar neutrophils. There were also rare foci of necrotic debris and alveolar hemorrhage (Fig 4A–E). The lungs of mice from the IN-pre group show near normal lung histopathology with only minimal perivascular mononuclear infiltrates (Fig 4F–J). The lungs of mice from the IP-pre group showed few perivascular mononuclear infiltrates, focal minimal alveolar hemorrhage, and occasional intra-alveolar neutrophils, whereas some areas also show near-normal lung histopathology (Fig 4K–O). The lung histopathology scores for mononuclear infiltrates, hemorrhage, PMN infiltrates, edema, and necrotic cellular debris were worse in the untreated infected group than in both the IP-pre and IN-pre–treated groups (Fig 4P). The differences in both pre-treated groups (IP-pre and IN-pre) as compared with infected untreated mice were highly significant for the main histopathologic findings: mononuclear infiltrates (*P* = 0.0297 and *P* = 0.0107, respectively) and alveolar hemorrhage (*P* = 0.0219 and *P* = 0.0165, respectively) (Fig 4P). The scores were better in the IN-pre group than the IP-pre group, but the difference did not reach statistical significance (Fig 4P).

**Figure S3.**
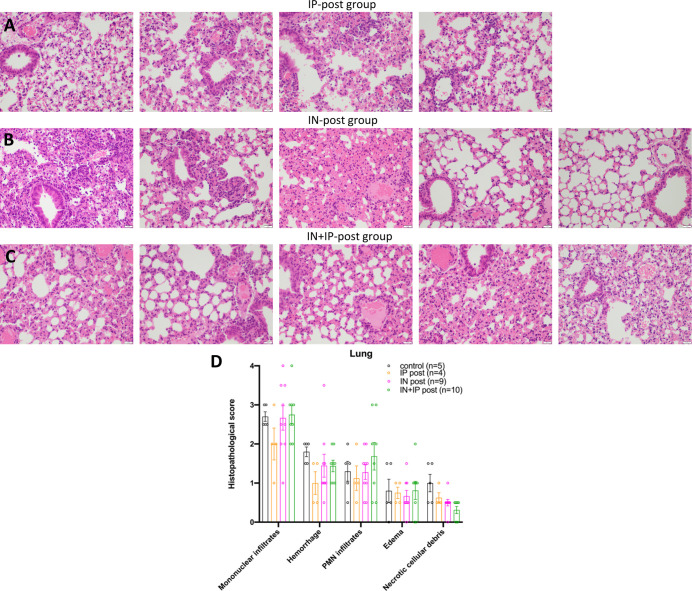
Representative examples of lung histopathology in H&E-stained lung slides of k18hACE2 mice inoculated with 2 × 10^4^ PFU SARS-CoV-2 that received ACE2 618-DDC-ABD post viral inoculation. **(A)** Four different mice from the group that received ACE2 618-DDC-ABD post-viral inoculation via IP show marked perivascular mononuclear infiltrates, alveolar hemorrhage, and scattered neutrophils and resemble mostly the infected untreated controls. **(B)** Five different mice from the group that received ACE2 618-DDC-ABD post-viral inoculation via IN also show marked perivascular mononuclear infiltrates, alveolar hemorrhage, and scattered neutrophils. In some mice from this group, however, lung histopathology was improved as compared with the infected untreated controls. **(C)** Five different mice from the group that received ACE2 618-DDC-ABD post-viral inoculation via IN + IP show marked perivascular mononuclear infiltrates, alveolar hemorrhage, and scattered neutrophils and are similar to the infected untreated controls. All photomicrographs were taken from H&E-stained sections at 40x magnification, scale bar = 500 μm. **(D)** The lung histopathology scores for mononuclear infiltrates, edema, and PMN infiltrates and edema in the infected untreated controls (black) are not different as compared with the post-treated mice with ACE2 618-DDC-ABD (color). The scores for hemorrhage necrotic cellular debris are lower in the treated groups (color) as compared with controls (black). These differences were not significant. Data shown as mean ± SEM. Significance was calculated by mixed-effects analysis followed by Tukey’s multiple comparisons test. Organs from one mouse each in the IP and IN group could not be obtained.

The lungs from the post-treated groups also showed perivascular mononuclear infiltrations and intra-alveolar neutrophils and resembled the infected untreated group (Fig S3A–C). In some mice from the IN-post and IN + IP-post group (Fig S3B and C), lung histopathology was improved but to a lesser extent than in the pre-treated groups (see Fig 4). The histopathological scores were not significantly different in infected untreated controls as compared with the post-treated groups (Fig S3D). Alveolar hemorrhage was reduced in the post-treated groups, but the difference was not statistically significant (Fig S3D).

### ACE2 618-DDC-ABD neutralizes WT and Omicron SARS-CoV-2 in two cell types

**Figure 5.**
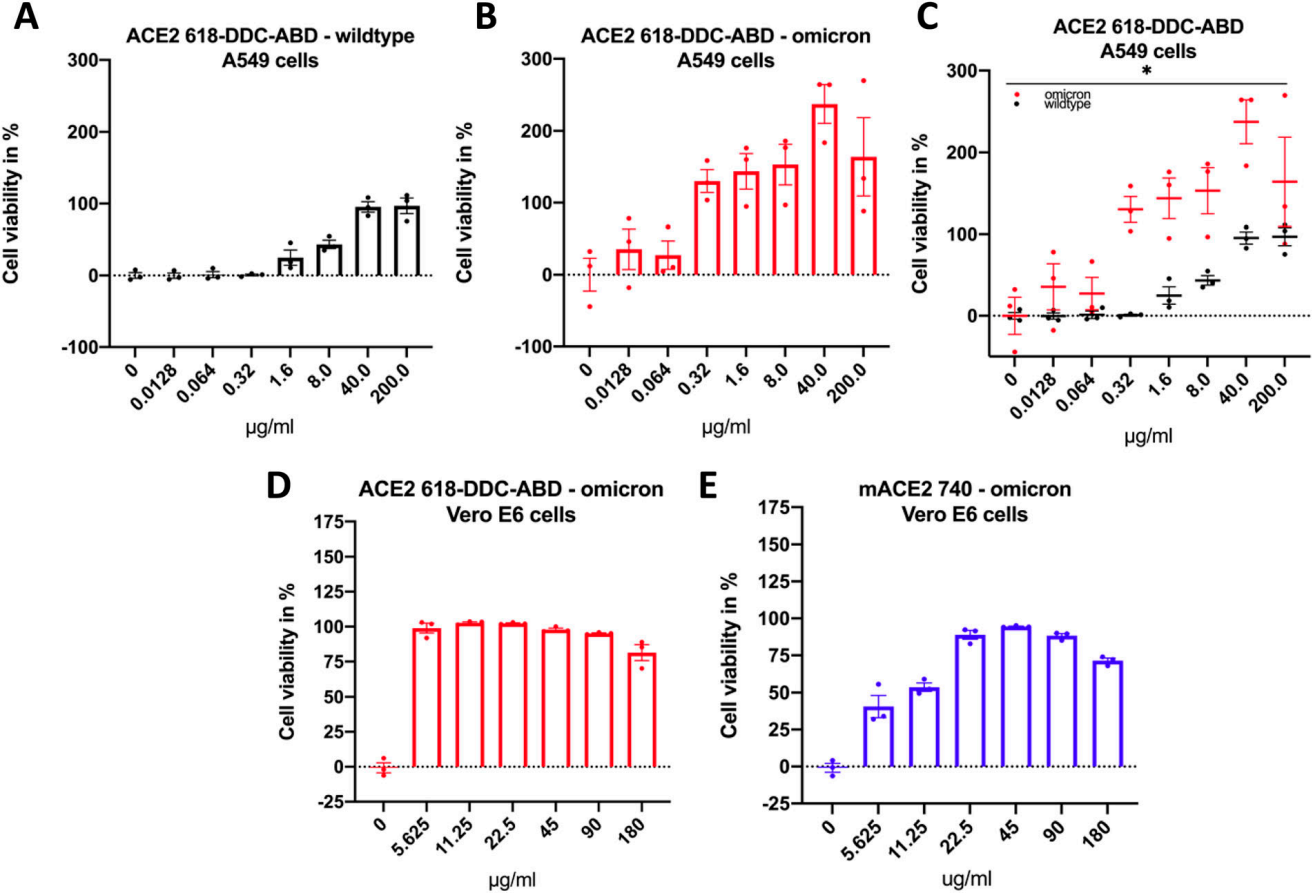
Neutralization of SARS-CoV-2 variants (500 PFU each) by human and mouse soluble ACE2 proteins after co-incubation for 1 h followed by infection of A549 or Vero E6 cells. **(A)** In A549 cells, human ACE2 618-DDC-ABD neutralizes WT SARS-CoV-2 at high concentrations (40 and 200 μg/ml), whereas lower concentrations (1.6 and 8 μg/ml) neutralize infection only partially and very low concentrations (0.0128–0.32 μg/ml) have no effect on infectivity. **(B)** In A549 cells, human ACE2 618-DDC-ABD neutralizes of the Omicron BA.1 variant of SARS-CoV-2 at concentrations lower than WT SARS-CoV-2 (0.32–200 μg/ml), and even very low concentrations have a partial effect. **(C)** The differences between WT (black) and Omicron BA.1 (red) neutralization by ACE2 618-DDC-ABD are significant (*P* = 0.0144, calculated by two-way ANOVA). **(D)** In Vero E6 cells, ACE2 618-DDC-ABD neutralizes the Omicron BA.1 variant at all concentrations tested (5.625–180 μg/ml). **(E)** In Vero E6 cells, mouse ACE2 740 neutralizes infection with the Omicron BA.1 variant but is only fully effective at concentrations of 22.5–180 μg/ml, whereas lower concentrations (5.625 and 11.25 μg/ml) have a partial effect. Values were normalized to the 0 μg/ml control and expressed as a percentage of mock (no virus) control wells. Mean ± SEM are shown.

In human A549 cells, ACE2 618-DDC-ABD neutralized WT SARS-CoV-2 (as shown by cell viability) at high concentrations (40 and 200 μg/ml). Lower concentrations (1.6 and 8 μg/ml) neutralized infection only partially, and very low concentrations (0.0128–0.32 μg/ml) had no effect on viral neutralization (Fig 5A).

By contrast, the Omicron BA.1 variant was neutralized by much lower concentrations of ACE2 618-DDC-ABD than WT SARS-CoV-2 (Fig 5B). When comparing the two sets of data (Omicron and WT variants), the difference was highly significant (*P* = 0.01) (Fig 5C).

This enhanced effect of ACE2 618-DDC-ABD on the neutralization of the Omicron variant in A549 cells was also found when we used Vero E6 cells, a nonhuman primate cell line that has been widely used for infectivity studies with SARS-CoV-2 (4, 8). In Vero E6 cells, ACE2 618-DDC-ABD neutralized the Omicron BA.1 variant at all concentrations tested (5.625–180 μg/ml) (Fig 5D).

The high sensitivity of this Omicron variant to ACE2 618-DDC-ABD prompted us to test a mouse soluble ACE2 protein that normally has no effect on WT SARS-CoV-2 infectivity (5). The mouse ACE2 740 protein neutralized Omicron BA.1 infection fully at high concentrations, whereas lower concentrations were only partially effective (Fig 5E).

## Discussion

The main finding of this study was that a soluble ACE2 protein, bioengineered to have extended duration of action and increased binding affinity for SARS-CoV-2, showed clear superiority of intranasal over systemic (intraperitoneal) administration in the k18hACE2 mouse model of SARS-CoV-2 infection. This superiority was shown by improvements in survival, clinical scores, and reduced lung viral titers. In the brains, moreover, the titers were undetectable in all animals in the group that received the treatment intranasally before viral inoculation. The soluble ACE2 protein, termed ACE2 618-DDC-ABD, was bioengineered to have increased duration of action by fusing a 618 amino acid truncate with an ABD and designed to have increased binding affinity to the S1 spike of SARS-CoV-2 by using a dodecapeptide (DDC) dimerization motif (8). Administration of this soluble ACE2 protein before viral inoculation, moreover, was far more effective regarding all the outcomes than administration only post-viral inoculation.

The k18hACE2 model in which human ACE2 transgene expression is driven by the k18 promoter is lethal after 5–7 d of inoculation with a high dose of WT SARS-CoV-2 (8, 10, 11, 14, 16, 17). The precise cause of the rapid lethality upon SARS-CoV-2 infection in this model is not fully understood. Brain SARS-CoV-2 titers in the infected untreated control group were one order of magnitude higher than that in the lungs (compare Fig 2A and B). The absence of brain titers in mice pre-treated intranasally explains, in our opinion, the much better survival than in untreated and groups treated with ACE2 618-DDC-ABD intraperitoneally. The group pre-treated by IN administration was indeed the only group in which brain viral titers were not detectable in any of the mice studied (Fig 2A). High brain viral titers, by contrast, were detected in the other groups with the exception of a few mice that survived until day 14 and, of note, these survivors had no brain viral titers detectable. Elevated brain viral titers, therefore, were associated with poor outcomes in terms of survival in both the pre- and post-viral inoculation groups. From these observations, we conclude that improved survival conferred by ACE2 618-DDC-ABD appears to be determined by two main factors: route of administration (IN better than IP) and timing (pre- and post-viral inoculation better than only post-viral inoculation).

Consistent with our data, previous studies had suggested that brain invasion of SARS-CoV-2 in k18hACE2 mice may be associated with more severe disease (18, 19). It should be pointed out, however, that brain injury was limited to only few animals in some studies (10, 14). Some of the findings that have been previously reported include encephalitis with leukocyte infiltration, hemorrhage, neuronal cell death, necrosis, and spongiosis (10, 14, 18, 19, 20, 39). In these previous studies with the k18hACE2 mice (10, 18, 19, 20, 39), however, no therapies were given. Therefore, the brain pathology could not be assessed regarding responses to therapies improving survival and organ protection. Here, we were able to show that survival conferred by the administration of our soluble ACE2 protein was associated with non-detectable brain viral titers. Consistent with the importance of viral brain invasion, in a study where k18hACE2 mice were inoculated intracranially with low doses of SARS-CoV, there was lethality despite little infection in the lungs (40). When SARS-CoV-2 was administered to k18hACE2 mice in aerosolized form for more direct lung delivery, and despite robust viral replication in the respiratory tract with airway obstruction, there was markedly reduced fatality and viral neuroinvasion (41). Our findings with intranasal delivery of soluble ACE2 before viral inoculation clearly demonstrate the importance of obliterating brain SARS-CoV-2 invasion for survival and brain protection.

We wish to point out, however, that the brain histopathologic findings were subtle even in untreated infected mice. The brain findings most frequently seen were perivascular and leptomeningeal lymphocytosis, endothelial hypertrophy, and parenchymal inflammation (Fig 3). These findings were mainly located in the striatum, cerebral cortex, and hypothalamus. Immunofluorescence for markers of microglia (IBA1) and astrocytes (GFAP) (42, 43) in the brain of infected untreated mice revealed high IBA1 and GFAP expression, consistent with reactive microgliosis and astrocytosis suggestive of an underlying neuroinflammatory state (Fig 3). Markers for astrocytes and microglia activation were also found in a study that examined cerebrospinal fluid from patients with severe Neuro-COVID-19 (44). In studies that used immunohistochemistry and imaging mass cytometry to examine brains from deceased COVID-19 patients, astrocyte and microglia activation was found as well (45, 46). In the IN group that received the treatment before viral inoculation, most brains appeared normal and IBA1 and GFAP expression was decreased, suggesting prevention of astrocyte and microglia activation and reduced neuroinflammation. Despite clear improvement in these parameters in mice treated with intranasal ACE2 618-DDC-ABD before viral inoculation, it remains to be determined how in brains from untreated infected mice viral invasion is associated with high mortality without more evident and severe histological damage.

Lung histopathology of the infected untreated mice showed dense perivascular mononuclear infiltrates, rare foci of alveolar hemorrhage and necrotic debris, and collections of intra-alveolar neutrophils. These findings were less pronounced in the IP-pre group and essentially absent in the IN-pre group which showed near-normal lung histopathology. It is very likely that in the IN-pre group the significantly improved lung histopathology was because SARS-CoV-2 lung titers were undetectable in half of the mice from this group (see Fig 2B). In the post-treated groups, lung histopathology was improved but not significantly different from the untreated infected controls; this suggests that a reduction in SARS-CoV-2 lung viral titers, if incomplete, could not fully prevent lung injury.

The mechanisms whereby soluble ACE2 proteins can neutralize SARS-CoV-2 have been previously discussed by us and others (3, 47). ACE2 exists in two forms: a full-length membrane bound form and a shorter soluble form that lacks the transmembrane domain (48, 49) and circulates in the blood in very small amounts (50). Both forms bind the receptor-binding domain of the SARS-CoV-2 S1 spike protein. By administering an abundant amount of soluble ACE2, the spike protein of SARS-CoV-2 can be intercepted from binding to the membrane bound ACE2 by the so-called decoy effect (3). To increase the binding affinity of ACE2 618-DDC-ABD to the receptor-binding domain of the SARS-CoV-2 S1 spike, a DDC motif was introduced that induces dimerization (8). By fusion with an ABD-tag, moreover, increased duration of action was achieved as demonstrated by its preserved plasma enzymatic activity for several days (5, 8). Membrane bound and soluble ACE2, including ACE2 618-DDC-ABD, metabolize angiotensin II and des-Arg^9^ bradykinin, two peptides that may be detrimental when accumulating (47, 51, 52, 53). This action may be especially beneficial in COVID-19 where internalization of ACE2–SARS-CoV-2 complexes causes depletion of cell membrane ACE2 which fosters accumulation of these pro-inflammatory peptides (53, 54, 55). Unfortunately, we were unable to measure these peptides because organ tissues could not be released from the BSL-3 facility. A high dose of soluble, enzymatically active form of ACE2 was well tolerated in the present study and, moreover, studies in normal mice not infected with SARS-CoV-2 showed that the administration of high doses of ACE2 do not lower blood pressure and has no effect on body weight or kidney function when given for months (56). In a safety and tolerability study in healthy human volunteers, systemic administration of a human soluble ACE2 18-740 (APN01) by intravenous injection was similarly well tolerated without causing hypotension or pulse rate disturbances (57). This is consistent with results obtained in preclinical pharmacological and toxicological investigations in rodents (58), piglets (59), and nonhuman primates, in which much higher doses of ACE2 18-740 (APN01) (up to 40 mg/kg) have been tested without any tolerability issues (57).

The membrane bound full-length ACE2 is essential for facilitating SARS-CoV-2 infection (6, 60). As shown by previous work with the soluble human ACE2 740 (6) and here with ACE2 618-DDC-ABD in two different permissive cell types, A549 and Vero E6 cells, high concentrations of soluble ACE2 are needed to neutralize infection of cells with WT SARS-CoV-2. Other variants of SARS-CoV-2, however, may be effectively treated with lower doses of soluble ACE2 proteins. This can be inferred from our findings in two different permissive cell lines, where ACE2 618-DDC-ABD neutralizes the Omicron BA.1 variant at lower protein concentrations (at least 20-fold lower than those required to neutralize WT SARS-CoV-2). It is also important to emphasize that soluble ACE2 protein–based approaches have universal effects against all the variants of SARS-CoV-2 (36). This is contrary to monoclonal antibodies that have the limitation of becoming less efficacious with each mutation of SARS-CoV-2, as consistently shown for the Omicron variants (23, 24, 25, 26, 27, 28, 29, 30, 31, 32, 33). Therefore, soluble ACE2 based therapies are likely to provide universal efficacy against all SARS-CoV-2 variants that evade monoclonal antibodies.

We conclude that ACE2 618-DDC-ABD provides better survival and organ protection when administered intranasally than systemically. Treatment post-viral inoculation, although less effective, still provides partially improved survival and organ protection. Abrogating brain SARS-CoV-2 invasion is a critical determinant of survival and organ protection in the k18hACE2 mouse model of lethal SARS-CoV-2 infection.

## Materials and Methods

### In vivo infectivity studies

All work with live SARS-CoV-2 in k18hACE2 mice was performed in the BSL-3 facility of the Ricketts Regional Biocontainment Laboratory, following a protocol approved by the IACUC of Northwestern University and University of Chicago. We used k18hACE2 mice that express full-length human ACE2 and are susceptible to SARS-CoV-2 infection (10, 14, 16, 17, 61, 62), purchased from Jackson Laboratory (8–13 wk old). Animals were infected intranasally with 2 × 10^4^ PFU SARS-CoV-2 in 20 μl (novel coronavirus/Washington/1/2020 was provided by N Thornburg [CDC] via the World Reference Center for Emerging Viruses and Arboviruses). Animals infected with this viral load invariably succumb to disease by days 5–9 (10, 14, 16, 17). We used different protocols to examine pretreatment and posttreatment effects of the soluble ACE2 618-DDC-ABD protein and to compare intranasal (IN) versus intraperitoneal (IP) administration effects. In the pretreatment groups, ACE2 618-DDC-ABD was administered to k18hACE2 mice (n = 10, five male and five female) via IN (30 μl, ∼13 μg/g BW) or via IP (200 μl, ∼13 μg/g BW) 1 h before SARS-CoV-2 followed by the same dose and 24 and 48 h later for a total of three doses. In the posttreatment groups, ACE2 618-DDC-ABD was administered either IN (30 μl, ∼12 μg/g BW, n = 10, male) or IP (200 μl, ∼1 μg/g BW, n = 5, male) or combined intranasally and intraperitoneally (IN + IP) (n = 10, male) 24, 48, and 72 h only post-viral inoculation (2 × 10^4^ PFU SARS-CoV-2). Controls (n = 5, male) received BSA in PBS both IN and IP at the same doses and time points as the ACE2 618-DDC-ABD post-treated animals.

Animals were weighed once daily and monitored twice daily for health using a clinical scoring system (Table S1). Animals that lost more than 20% of their baseline body weight or had a clinical score of three were euthanized for humane reasons (humanely euthanized) and considered a fatal event as per study protocol. Mice were euthanized by using CO_2_-forced inhalation. After the last breathing movement, cervical dislocation was performed to prevent the mice from recovering from CO_2_ exposure. To be able to compare viral titers and organ pathology at the same time point, randomly selected animals from the IN group (which all appeared healthy based on clinical score) were euthanized on day 5 together with the animals from the IP group that were euthanized because the mortality endpoint was reached. Otherwise, animals that did not reach the severity of these criteria were monitored for up to 14 d in the BSL-3 facility and euthanized at day 14.

Table S1. Scoring system in BSL-3 facility for health evaluation of mice infected with SARS-CoV-2.

Portions of lungs and brains were removed from all euthanized animals and were used for viral load measurements by plaque assay (see below), whereas the remaining portions were fixed in 10% formalin and embedded in paraffin for histopathology and immunostaining. The Mouse Histology and Phenotyping Laboratory center at Northwestern University generated slides for staining studies.

Hematoxylin and eosin (H&E)–stained sections were evaluated by expert lung pathologists on a scoring system, recently described for SARS-CoV-2–infected k18hACE2 mice (8, 14). The categories scored were: mononuclear infiltrates, alveolar hemorrhage, edema, cellular necrosis, hyaline membranes, and thrombosis. The scale was as follows: 0 = no detection, 1 = uncommon detection in <5% lung fields (200x), 2 = detectable in up to 30% of lung fields, 3 = detectable in 33–66% of lung fields, and 4 = detectable in >66% of lung fields. Neutrophil infiltration was evaluated on a scale of 0–3 as follows: 0 = within normal range, 1 = scattered PMNs sequestered in septa, 2 = score 1 and solitary PMNs extravasated in airspaces, 3 = score 2 plus and aggregates in vessel and airspaces.

Brain injury was evaluated on H&E-stained sections by a blinded neuropathologist and scored for leukocytosis and lymphocytosis and neuronal pyknosis on a scale of 0–3. The scale was as follows: 0 = none, 1 = mild (focal), 2 = moderate (multifocal), 3 = severe (diffuse).

### ACE2 enzymatic activity in the brain

In pilot studies in non-infected mice, ACE2 618-DDC-ABD was administered intranasally to see if it reaches the brain. For this, ACE2 deficient mice (total body ACE2/PRCP double-knockout mice) (50) were given ACE2 618-DDC-ABD protein intranasally (10 μg/g BW, 35 μl total volume in both nostrils) under general ketamine–xylazine anesthesia. The animals recovered from anesthesia and 4 h after intranasal instillation were euthanized by overdose of Euthasol. Mice were perfused with PBS to flush out blood from the organs. Brains were then removed and tissue lysates obtained by homogenization in RIPA buffer (63). The lysates were then clarified by centrifugation at 6,000*g* for 10 min at 4°C. Protein concentration in the cleared lysates was measured using BCA assay kit (Thermo Fisher Scientific). The cleared tissue lysates were diluted in a 1x TBS, pH 7.4 (cat#BP2471-1; Thermo Fisher Scientific). For ACE2 activity, a fluorogenic substrate Mca-APK-Dnp (Bachem) was used, and the plates were read using a fluorescence plate reader FLX800 (BioTek Instruments) at an excitation wavelength of 320 nm and an emission wavelength of 400 nm. All reactions were performed at ambient temperature in microtiter plates with a 100 μl total volume. Each sample was tested in duplicate wells with one of the two wells used as a blank. A specific inhibitor of ACE2 (MLN-4760, gift from Millennium Pharmaceuticals) was used at 10^−5^ M end concentration (64, 65) in the blank wells. ACE2 activity (relative fluorescence units) was calculated by subtracting blank values from values of wells without ACE2 inhibitor and divided by total protein concentration of the tissue lysates. ACE2 enzymatic activity in brain lysates was not detectable in ACE2KO mice that received PBS but was detectable in those infused with ACE2 618-DDC-ABD (0.004 ± 0.0002 RFU/μg protein/hr and 0.14 ± 0.098 RFU/μg protein/h, *P* = 0.015).

### Plaque assay for infectious virus

Tissue samples were collected in DMEM with 2% FBS and were homogenized using 1.4 mm ceramic beads in a tissue homogenizer using two 30 s pulses. Samples were then centrifuged at 1,000*g* for 5 min, and the supernatant was serially diluted 10-fold and used to infect Vero E6 cells for 1 h. Inoculum was removed, and 1.25% methylcellulose DMEM was added to the cells and incubated for 3 d. Plates were fixed in 1:10 formalin for 1 h and stained with crystal violet for 1 h and counted to determine PFU and expressed as PFU/ml after the data were normalized by organ weight.

### Immunofluorescence

For immunofluorescence staining studies of the brain, IBA1 (ab178846; Abcam) and GFAP (ab4674; Abcam) antibodies were used.

### SARS-CoV-2 infection of A549 and Vero E6 cells

All work with live SARS-CoV-2 was performed in hACE2-A549 or Vero E6 cells in the BSL-3 facility of the Ricketts Regional Biocontainment Laboratory. 500 PFU of each SARS-CoV-2 strain: WT (novel coronavirus/Washington/1/2020 was provided by N. Thornburg [CDC] via the World Reference Center for Emerging Viruses and Arboviruses) or Omicron BA.1 (BEI NR-56481, obtained through BEI Resources, NIAID, NIH: SARS-related coronavirus 2, Isolate hCoV-19/USA/GA-EHC-2811C/2021 [Lineage B.1.1.529; Omicron variant], NR-56481, contributed by Mehul Suthar) of SARS-CoV-2 was incubated with various concentrations (0.0128, 0.064, 0.32, 1.6, 8.0, 40.0, 200 μg/ml for A549 cells or 5.626, 11.25, 22.5, 45, 90, 180 μg/ml for E6 cells) of the different soluble ACE2 proteins (human ACE2 618-DDC-ABD or mouse ACE2 740) for 1 h at 37°C. This mixture was then used to infect the respective cell types. Cells were then incubated for 3–4 d (WT SARS-CoV-2) or 5 d (Omicron BA.1) until a noticeable cytopathic effect was observed in control wells (0 μg/ml of soluble ACE2 proteins). Cell numbers were assessed by staining cells with crystal violet and reading absorbance of each well at 595 nm. Values were then normalized to the 0 μg/ml control and expressed as a percentage of mock (no virus) control wells.

### Statistics

GraphPad Prism v8.4.3 (GraphPad Software) was used to calculate statistics. Normality was tested using the Shapiro–Wilk test. Differences between more than two groups with normally distributed data were analyzed by ANOVA followed by post hoc Dunnett’s multiple comparisons test. Differences between more than two groups with non-normally distributed data were analyzed by the Kruskal–Wallis test followed by the post hoc Dunn’s multiple comparisons test. Differences between two groups with normally distributed data were analyzed by unpaired *t* test. Differences between two groups with non-normally distributed data were analyzed by Mann–Whitney test. Differences in survival were calculated by log-rank (Mantel–Cox) test.

### Study approval

All work with live SARS-CoV-2 in k18hACE2 mice was performed in the BSL-3 facility of the Ricketts Regional Biocontainment Laboratory, following a protocol approved by the Institutional Animal Care and Use Committees of both Northwestern University (IS00004795) and University of Chicago (72642).

## Supplementary Material

Reviewer comments
